# Health care management of sickness certification tasks: results from two surveys to physicians

**DOI:** 10.1186/1756-0500-6-207

**Published:** 2013-05-23

**Authors:** Christina Lindholm, Mia von Knorring, Britt Arrelöv, Gunnar Nilsson, Elin Hinas, Kristina Alexanderson

**Affiliations:** 1 Department of Clinical Neuroscience, Division of Insurance Medicine, Karolinska Institutet, Stockholm, Sweden; 2 Stockholm County Council, Stockholm, Sweden; 3 Department of Neurobiology, Care Sciences and Society, Centre for Family and Community Medicine, Karolinska Institutet, Stockholm, Sweden; 4 Department of Learning, Informatics, Management and Ethics (LIME) Karolinska Institutet, Stockholm, Sweden

**Keywords:** Health care management, Sickness certification practice, Sick leave, Physician

## Abstract

**Background:**

Health care in general and physicians in particular, play an important role in patients’ sickness certification processes. However, a lack of management within health care regarding how sickness certification is carried out has been identified in Sweden. A variety of interventions to increase the quality of sickness certification were introduced by the government and County Councils. Some of these measures were specifically aimed at strengthening health care management of sickness certification; e.g. policy making and management support. The aim was to describe to what extent physicians in different medical specialties had access to a joint policy regarding sickness certification in their clinical settings and experienced management support in carrying out sickness certification.

**Method:**

A descriptive study, based on data from two cross-sectional questionnaires sent to all physicians in the Stockholm County regarding their sickness certification practice. Criteria for inclusion in this study were working in a clinical setting, being a board-certified specialist, <65 years of age, and having sickness certification consultations at least a few times a year. These criteria were met by 2497 physicians in 2004 and 2204 physicians in 2008. Proportions were calculated regarding access to policy and management support, stratified according to medical specialty.

**Results:**

The proportions of physicians working in clinical settings with a well-established policy regarding sickness certification were generally low both in 2004 and 2008, but varied greatly between different types of medical specialties (from 6.1% to 46.9%). Also, reports of access to substantial management support regarding sickness certification varied greatly between medical specialties (from 10.5% to 48.8%). More than one third of the physicians reported having no such management support.

**Conclusions:**

Most physicians did not work in a clinical setting with a well-established policy on sickness certification tasks, nor did they experience substantial support from their manager. The results indicate a need of strengthening health care management of sickness certification tasks in order to better support physicians in these tasks.

## Background

Physicians play an important role in the process involving sickness absence; diagnosing, treating, and suggesting rehabilitation measures regarding the patients’ medical problems and, in their role as medical experts, issuing a medical certificate concerning functional impairment and work capacity [1-3].

Several studies have shown that physicians experience sickness certification as problematic [1,4-17]. Lack of management within the health care organization regarding the tasks that are involved has been reported [9,10,18]; however, very few studies have focused on this issue [19,20]. In a review of European health management research between 1995 and 2005, including studies on policy development and clinical management [21], no studies on management of sickness certification tasks were reported.

In Sweden, the National Board of Health and Welfare, which is the supervisory government agency, has stated that sickness certification is an aspect of health care and medical treatment and, as such, is to be managed according to the same laws and regulations as other types of medical treatment. This includes managerial responsibility to regularly and systematically develop and assure the quality of the health services provided and to organize health services so that safe and good care for patients can be delivered [22,23].

In 2003, the Board for the first time ever investigated how sickness certification was dealt with in health care and concluded that there was a lack of management of the tasks involved, including of quality assurance, e.g. regarding documentation, and also that most clinical settings lacked a required policy or guidelines regarding how to handle sickness certification of patients. Furthermore, a comprehensive investigation conducted for the Swedish government regarding problems in how health care handled sickness certification, established that there was a genuine lack of such management; at no organizational level within health care was management of sick-listing tasks on the agenda. Overall, there were no strategies for quality assurance of how the tasks were performed, for developing competence in performing these tasks, for how to cooperate with others within and outside healthcare, or for generating knowledge within the area [10,23].

Based on findings from the above reports, as well as on the urgent need to deal with the sick-leave rates in Sweden, which were very high from an international perspective [24,25], the government and other stakeholders, such as the Social Insurance Agency and the County Councils, initiated several measures. Stockholm County, which is by far the largest County Council in Sweden, initiated several interventions directed toward increasing the management of sickness certification tasks. Several of these measures began in 2007, including introduction of a regional policy for sickness certification and audits aimed at developing sickness certification policies at the clinical setting level. Some activities were directed towards managers, focusing on their role and responsibility in the sickness certification process. Other measures were directed towards physicians and most of them towards GPs.

Management includes a number of elements at different levels in an organization and consequently there are several ways to investigate these issues. In this study, the focus was on management in different medical specialties, and we chose, in 2004 and 2008, to ask physicians in the different medical specialties about the presence of a policy, and of management support regarding sickness certification.

The aim of this study was to describe to what extent physicians in different medical specialties had access to a joint policy regarding sickness certification at their place of work and if they experienced management support in sickness certification tasks.

## Methods

Data from two cross-sectional surveys were analyzed. Questionnaires were sent to physicians in 2004 [15] and in 2008 [16]. The first survey included the 7665 physicians who were below 65 years of age and worked either in the Stockholm County or Östergötland County [15]. The age limit was used as 65 is the common age for old-age pension in Sweden. The physicians in Stockholm were identified through their membership in the Swedish Medical Association, through which they were registered as working and living in Stockholm in 2004. About 95% of the physicians in Sweden were members of that association. The 2008 survey included all 36 898 physicians living and working in Sweden in October 2008. They were identified using a register of all physicians in Sweden that was maintained by Cegedim AB, a Swedish company [16].

A comprehensive questionnaire about various aspects of sickness certification practice and related work tasks was developed in 2004 (83 questions) [15]. This questionnaire was further developed in 2008 (163 questions) [16]. The questionnaires were sent by mail in October 2004 and October 2008, respectively, to the participants’ home addresses in order to avoid interaction with colleagues in completing the questionnaire. Two and three reminders, respectively, were sent to non-responders in 2004 and 2008. Distribution, registration, scanning of questionnaires, and basic management of data was administered by Statistics Sweden.

Of the physicians who had answered the questionnaire in 2004 or 2008, the following were included in this study: those who were board-certified specialists, worked mainly in a clinical setting in the Stockholm County, were below 65 years of age, and responded that they had consultations regarding sickness certification a few times a year or more (the five response options ranged from >10 times per week to never). The study population comprised 6794 physicians in 2004 and 9391 physicians in 2008. The response rates were 71% in 2004 and 57% in 2008 (Table 1). In all, 2497 physicians fulfilled the inclusion criteria in the 2004 survey and 2204 in the 2008 survey.

**Table 1:** The number and percentages of the study population, the responders, and the study group in 2004 and 2008, respectively

	**Year**	**Study population**	**Responding physicians**		**Board-certified specialists with sickness certification tasks**
		**n**	**n**	**%**	**n**
All	2004	6794	4827	71.0	2497
	2008	9391	5369	57.2	2204
Women	2004	-	2464	**74.0**	1200
	2008	4651	2840	61.1	1077
Men	2004	-	2363	**68.0**	1297
	2008	4738	2529	53.4	1127

Response rates in bold are the combined rates for Stockholm and Östergötland County.

### Data

Information about age, sex, and board-certificated specialty was provided by the National Board of Health and Welfare. Questionnaire information concerned the main clinical settings of the physicians in the following medical specialties; rehabilitation, oncology, occupational health services, orthopedics, internal medicine, gynecology, surgery, primary care, and psychiatry. All other medical specialties were combined into the “other medical specialties” group.

Management of the physicians’ sickness certification tasks was assessed by the following two questions:

1. “Is there a joint policy where you work for handling matters related to sickness certification tasks?” The response alternatives in 2004 were: “Yes, and it is well established”, “Yes, to some extent”, and “No”. In 2008, two additional response alternatives were included: “I don’t know” and “Not applicable, I don’t work in a clinical setting”. Those who selected the last response alternative were not included in the study.

2. ‘Do you have support from your manager regarding sickness certification cases?’ In 2004 the response alternatives were: “Yes, extensive support”, “Yes, some support”, and “No”. In 2008 there were two additional response alternatives: “Not applicable, I don’t have a manager” and “Not applicable, I don’t work in a clinical setting”.

The partial non-response rates (missing data in returned questionnaires) for these two questions were 4.2% and 8.0%, respectively, in 2004, and 0.9% and 5.0%, respectively, in 2008.

### Statistical analysis

Results from descriptive statistics for frequencies regarding the two questions in 2004 and 2008 were stratified by type of medical specialty and the proportions, with 95% confidence intervals (CI), for giving the different response alternatives were calculated for each type of medical specialty using the SPSS 18.0 program.

To check whether the two items regarding policy and support, respectively, measured the same aspects of management or could be regarded as capturing somewhat different aspects, we calculated Pearson’s correlation coefficient between having a well-established policy (well established/all other) and having substantial support from a manager in this task (substantial support/all other). Correlation between the two items assessing management was generally weak (r = 0.40 in 2004 and r = 0.25 in 2008), indicating that they capture different aspects of management.

### Ethics

This study was approved by the Regional Ethical Review Board of Stockholm, Sweden. The Board found no ethical obstacles, based on the Declaration of Helsinki, to carrying out the study (Dnr 04-315/1 and Dnr 2008/795-31).

## Results

**Figure 1:**
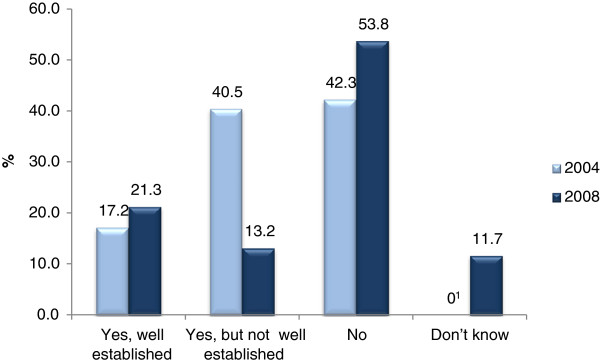
**Proportions (%) of physicians having a sickness certification policy in 2004 and 2008, respectively. **^1^The response alternative "Don´t know" was not included in the 2004 questionnaire.

In 2004, 57.7% of the physicians worked in a clinical setting with a joint policy regarding sickness certification; 17.2% stated that the policy was well established (Figure 1). In 2008, a lower proportion (34.5%) stated that they had such a policy and among them 21.3% had a well established policy. The variation among medical specialties regarding access to a well-established policy was substantial in both surveys, ranging from 6.1% among physicians in internal medicine to 41.5% in rehabilitation medicine in 2004 and from 8.8% in internal medicine to 46.9% in occupational health service in 2008 (Table 2). Specialists in rehabilitation medicine clinics and in occupational health services had the highest rates both years, however, with wide CIs. The proportions of physicians stating having a well established policy were about the same the two years, however, the proportion of physicians stating ‘no’ (policy) were higher in 2008 except for rehabilitation specialists. The proportion of GPs stating having a well-established policy was 12.8% in 2004 and 26.8% in 2008. Compared to in 2004 a higher proportion of specialists in gynecology, psychiatry, and primary care stated 2008 that they had no joint policy regarding sickness certification. However, some of the participants might be the same 2004 and 2008 while others have changed specialty and work site.

**Table 2:** Physicians (n and %) in different medical specialties who reported having a sickness certification policy in 2004 and in 2008, respectively

**Medical specialty**	**Year**	**Physicians, n**	**Do you and your colleagues in your clinical setting have a joint policy for handling matters related to sickness certification?**							
			**Yes, well established % (95% CI)**		**Yes, but not well established % (95% CI)**		**No % (95% CI)**		**Do not know % (95% CI)**	
Rehabilitation	2004	41	41.5	(25.7-57.2)	22.0	(8.7-35.2)	36.6	(21.2-52.0)	-	-
	2008	39	46.2	(29.8-62.5)	23.1	(9.2-36.9)	23.1	(9.2-36.9)	7.7	(−1.1-16.4)
Oncology	2004	57	12.3	(3.5-21.1)	40.4	(27.2-53.5)	47.4	(34.0-60.7)	-	-
	2008	60	18.3	(8.3-28.4)	10.0	(2.2-17.8)	55.0	(42.0-68.0)	16.7	(7.0-26.4)
Occupational	2004	98	33.7	(24.1-43.2)	38.8	(29.0-48.6)	27.6	(18.5-36.6)	-	-
health service	2008	96	46.9	(36.7-57.0)	12.5	(5.8-19.2)	37.5	(27.6-47.4)	3.1	(−0.4-6.7)
Orthopedics	2004	109	10.1	(4.3-15.8)	38.5	(29.2-47.8)	51.4	(41.8-60.9)	-	-
	2008	103	10.7	(4.6-16.7)	16.5	(9.2-23.8)	66.0	(56.7-75.3)	6.8	(1.9-11.7)
Internal	2004	164	6.1	(2.4-9.8)	30.5	(23.4-37.6)	63.4	(56.0-70.9)	-	-
medicine	2008	238	8.8	(5.2-12.5)	8.4	(4.9-12.0)	64.7	(58.6-70.8)	18.1	(13.1-23.0)
Surgery	2004	168	10.7	(6.0-15.4)	36.9	(29.5-44.3)	52.4	(44.8-60.0)	-	-
	2008	214	15.4	(10.5-20.3)	15.9	(11.0-20.8)	58.4	(51.8-65.1)	10.3	(6.2-14.4)
Gynecology	2004	171	20.5	(14.4-26.6)	51.5	(43.9-59.0)	28.1	(21.3-34.9)	-	-
	2008	150	18.0	(11.8-24.2)	18.0	(11.8-24.2)	50.0	(41.9-58.1)	14.0	(8.4-19.6)
Psychiatry	2004	264	20.1	(15.2-24.9)	40.5	(34.6-46.5)	39.4	(33.5-45.3)	-	-
	2008	214	19.6	(14.3-25.0)	9.3	(5.4-13.3)	52.8	(46.1-59.5)	18.2	(13.0-23.4)
Primary	2004	555	12.8	(10.0-15.6)	55.0	(50.8-59.1)	32.3	(28.4-36.2)	-	-
care	2008	522	26.8	(23.0-30.6)	18.2	(14.9-21.5)	49.4	(45.1-53.7)	5.6	(3.6-7.5)
Other medical	2004	764	20.5	(17.7-23.4)	31.9	(28.6-35.3)	47.5	(44.0-51.1)	-	-
specialties	2008	549	21.3	(17.9-24.7)	8.9	(6.5-11.3)	55.6	(51.4-59.7)	14.2	(11.3-17.1)
All specialists	2004	1836	18.6	(16.8-20.4)	36.1	(33.9-38.3)	45.3	(43.0-47.6)		
*except* GPs	2008	1663	19.5	(17.6-21.5)	11.7	(10.1-13.2)	55.2	(52.8-57.6)	13.5	(11.9-15.1)
All specialists	2004	2391	17.2	(15.7-18.7)	40.5	(38.5-42.5)	42.3	(40.3-44.3)	-	-
	2008	2185	21.3	(19.6-23.0)	13.2	(11.8-14.6)	53.8	(51.7-55.9)	11.7	(10.3-13.0)

The percentages (%) are calculated with 95% confidence intervals (CI) by medical specialty. The response alternative ‘Do not know’ was not included in the 2004 questionnaire.

The rates of missing responses were 4.2% (n = 106) in 2004 and 0.9% (n = 19) in 2008.

The proportion of physicians with substantial management support (Table 3) was 25.3% and 18.1% in 2004 and in 2008, respectively. The variation among medical specialties was about as wide as for having a well-established policy; 13.7% in internal medicine and 48.8% in rehabilitation medicine in 2004 and 10.5% in oncology and 34.2% in rehabilitation medicine in 2008.

**Table 3:** Physicians’ (n and % with 95% confidence intervals (CI)) experience of management support by medical specialty in 2004 and 2008, respectively

**Medical specialty**	**Year**	**Physicians (n)**	**Do you have support from your manager regarding handling of sickness certification cases?**							
			**Yes to a great extent % (95% CI)**		**To some extent % (95% CI)**		**No % (95% CI)**		**Do not have a manager % (95% CI)**	
Rehabilitation	2004	41	48.8	(32.8-64.8)	22.0	(8.7-35.2)	29.3	(14.7-43.8)	-	-
	2008	38	34.2	(18.4-50.0)	31.6	(16.1-47.1)	21.1	(7.5-34.6)	13.2	(1.9-24.4)
Oncology	2004	56	17.9	(7.5-28.2)	32.1	(19.5-44.8)	50.0	(36.5-63.5)	-	-
	2008	57	10.5	(2.3-18.7)	42.1	(28.9-55.3)	45.6	(32.3-58.9)	1.8	(−1.8-5.3)
Occupational	2004	94	41.5	(31.3-51.6)	19.1	(11.0-27.3)	39.4	(29.3-49.4)	-	-
health service	2008	97	29.9	(20.6-39.2)	33.0	(23.5-42.5)	25.8	(16.9-34.6)	11.3	(4.9-17.8)
Orthopedics	2004	105	27.6	(18.9-36.3)	34.3	(25.1-43.5)	38.1	(28.7-47.5)	-	-
	2008	102	13.7	(6.9-20.5)	38.2	(28.6-47.8)	37.3	(27.7-46.8)	10.8	(4.7-16.9)
Internal	2004	161	13.7	(8.3-19.0)	44.1	(36.3-51.9)	42.2	(34.5-49.9)	-	-
medicine	2008	227	14.5	(9.9-19.2)	34.4	(28.1-40.6)	42.7	(36.2-49.2)	8.4	(4.7-12.0)
Gynecology	2004	164	26.8	(20.0-33.7)	39.6	(32.1-47.2)	33.5	(26.2-40.8)	-	-
	2008	143	22.4	(15.5-29.3)	38.5	(30.4-46.5)	24.5	(17.3-31.6)	14.7	(8.8-20.6)
Surgery	2004	164	20.1	(13.9-26.3)	34.8	(27.4-42.1)	45.1	(37.4-52.8)	-	-
	2008	204	16.7	(11.5-21.8)	29.9	(23.6-36.2)	40.2	(33.4-47.0)	13.2	(8.5-17.9)
Psychiatry	2004	248	25.4	(19.9-30.9)	38.3	(32.2-44.4)	36.3	(30.3-42.3)	-	-
	2008	203	19.2	(13.7-24.7)	37.9	(31.2-44.7)	31.0	(24.6-37.5)	11.8	(7.3-16.3)
Primary	2004	546	29.5	(25.7-33.3)	44.5	(40.3-48.7)	26.0	(22.3-29.7)	-	-
care	2008	514	21.4	(17.8-25.0)	41.6	(37.4-45.9)	25.1	(21.3-28.9)	11.9	(9.1-14.7)
Other medical	2004	718	22.1	(19.1-25.2)	35.0	(31.5-38.5)	42.9	(39.3-46.5)	-	-
specialties	2008	509	13.4	(10.4-16.3)	33.6	(29.5-37.7)	35.2	(31.0-39.3)	17.9	(14.5-21.2)
All specialists	2004	1751	23.9	(21.9-25.9)	35.4	(33.2-37.7)	40.7	(38.4-43.0)		
*except* GPs	2008	1580	17.0	(15.1-18.8)	34.7	(32.4-37.1)	35.0	(32.6-37.4)	13.3	(11.6-15.0)
All specialists	2004	2297	25.3	(23.5-27.0)	37.6	(35.6-39.6)	37.2	(35.2-39.2)	-	-
	2008	2094	18.1	(16.4-19.7)	36.4	(34.4-38.5)	32.6	(30.6-34.6)	12.9	(11.5-14.4)

The response alternative ‘Do not have a manager’ was not included in the 2004 questionnaire.

Rates of missing answers for this item were 8.0% (n = 200) in 2004 and 5.0% (n = 110) in 2008.

The proportions of physicians experiencing *no* managerial support were about the same in both surveys, both for all and in different specialties, with oncology and surgery having the highest rates. For both aspects of management, physicians in rehabilitation medicine had the highest proportion in both the surveys (Tables 2 and 3). However, the CIs were very wide.

## Discussion

In this study, we investigated two aspects of health care management of sickness certification tasks using survey data from physicians, namely: physicians’ access to a joint policy and their access to management support in two different years. In both surveys, the majority of the physicians reported not having substantial management support regarding sickness certification tasks and that their clinical setting lacked a well-established policy regarding these tasks – something that is warranted according to rules of Swedish healthcare. However, there were considerable differences between medical specialties regarding these two aspects of management.

The results are based on two cross-sectional surveys and cannot provide empirical bases for explanations of differences between the specialists’ responses the two years. The large differences between different specialists experiences of managerial support and work-place policy regarding sickness certification tasks can however be discussed in different ways. One possible hypothesis is that in some type of clinics, a larger share of the patients has more diffuse symptoms and a higher need of sickness certification, which may result in higher needs of managerial support or policy. Another possible hypothesis about the differences might be to what extent there has been a focus on aspects of sickness certification in the various specialties and clinics.

Regarding the differences and similarities between the two years there is a need of caution in interpreting the results. We do not know if the participants worked at the same clinic in 2008 as in 2004, nor how many that participated in only one of the survey. Differences in proportions, at an organizational level, can be due to other people answering, to changes in management, or to changes in how physicians understand the need of management, operationalised in these two measures of management.

Physicians’ responses to questions about management’s support of sickness certification processes are also likely influenced by the context they work in. However, the many interventions taken in Stockholm County from 2006 cannot be expected to have influenced these aspects much, given the short time frame. Implementation of change in health care organizations has been described in management theories [1,26,27] as a slow process. Furthermore, there is still limited understanding about how to develop organizations as well as about how to influence organizational changes [28,29]. According to previous research, management is rather weak in relation to physicians, and the lack of management many physicians experienced in this study might reflect the difficulty in implementing change and communicating among the various levels of policymaking, managers, and physicians within the health care organization [30-33]. Future studies are warranted about these aspects.

Due to a lack of other empirical studies in this area, we cannot assess whether these results are in line with other studies or to what extent access to policy and managerial support cover the health care management of sickness certification tasks.

### Strengths and limitations

The main strengths of the study were the large number of participants, permitting sub-group analyses regarding different medical specialties, and that all physicians in Stockholm County, not just a sample, were included. Only specialists were included in the analysis, which means that the participants had experience concerning the aspects being studied. Excluding non-specialists and those not mainly working in clinical settings, also reduced plausible bias for differences in experienced management support and policy between medical specialties with high and low proportions of specialists. Moreover, the low correlations in how the participants had responded to the two items indicate that the two items cover two different aspects of management.

Another strength was the relatively high response rates, considering the often low rates among physicians [11,34,35]. Nevertheless, there were substantial rates of non responders, and a great limitation is that we have no way of knowing how those would have responded.

Moreover, the response rate was lower in 2008 (57% 2008 and 71% 2004), possibly partly due to the expanded questionnaire, from 83 to 163 questions. Another limitation is the difference in response alternatives between the two surveys and this might be one explanation for the results showing a lower rate of management support in 2008. Hence, we have not made any comparisons between the surveys to avoid such bias.

The inclusion of additional response alternatives in 2008 was based on responders’ open comments to the 2004 survey, showing a need for more detailed alternatives to increase the validity of the 2008 responses. Supplementary analyses excluding physicians who answered “I don’t have a manager” (added in 2008) did not alter the results. Also, we do not know to what extent the same physicians participated in the two questionnaires.

## Conclusions

Most physicians did not work in a clinical setting with a well-established policy on sickness certification tasks, nor did they experience substantial support from their manager. The results indicate a need of strengthening health care management of sickness certification tasks in order to better support physicians in these tasks.

## Competing interests

The authors declare that they have no competing interests.

## Authors’ contributions

CL, BA, GN, and KA participated in the design of the study, data collection, and interpretation of data, and drafted the manuscript. MvK and EH contributed to the interpretation of data and drafted the manuscript. EH performed the statistical analyses. All authors have read and approved the final manuscript.
